# Incorporating topical stance into signed bipartite networks for user retweet prediction

**DOI:** 10.1371/journal.pone.0342677

**Published:** 2026-02-23

**Authors:** Lixia Li, Ziqing Chen, Haoran Ye, Yixuan Zhang

**Affiliations:** 1 Wuhan Digital Engineering Institute, Wuhan, China; 2 Electronic Information School, Wuhan University, Wuhan, China; PLOS: Public Library of Science, UNITED KINGDOM OF GREAT BRITAIN AND NORTHERN IRELAND

## Abstract

Social networks accelerate information dissemination, and retweet behavior is an important way of user interaction. User retweet prediction analyzes user characteristics to predict retweet behavior and emotional polarity, which can help platforms understand user emotional tendencies and can be applied to scenarios such as public opinion analysis. However,most existing studies on tree like propagation chains focus on link existence and rarely combine positive negative polarity with topic semantics. This paper constructs a signed bipartite network using user and topic nodes, proposes a Topic Node weighting-based Topical Stance Representation (TNTSR) method, and develops a Topic Stance-integrated Graph Attention Neural Network (TSGAT) for retweet prediction. Experiments on social platform datasets show that both methods outperform benchmarks like SGCN in predicting retweet behavior and polarity. This research effectively leverages topic stance and network structure, enhancing the accuracy of user retweet prediction.

## Introduction

User retweet behavior in social networks is an important means of information dissemination and interaction, with retweet relationships and emotions in texts holding significant research value [1–3]. The core goal of user retweet prediction is to construct models that accurately predict information propagation paths and influence by analyzing user characteristics, social network structures, and content attributes. This not only helps platforms understand user emotional tendencies but also applies to practical scenarios such as public opinion analysis [4–6], advertising marketing [7], and crisis management [8,9].

In recent years, researchers have conducted extensive studies on user retweet prediction in social networks [10–12]. However, existing studies do not consider features such as relationships between users and individual preferences. In real world social networks, constrained by platform limitations and self interests, users do not directly reveal polar relationships (especially negative ones) with others. The relationship between users is usually determined by their attitude, emotions, and attitudes towards the topic. For example, users’ opinion stances on public opinion topics. Therefore, when user relationships are missing, it is necessary to reconstruct the user network structure and edge polarity based on users’ stances toward topics. However, existing methods ignore the information contained in the topic nodes during reconstruction, leading to inaccurate characterization of user relationships and affecting the analysis of user stances and the prediction accuracy of user retweet behavior.

Existing approaches suffer from a critical limitation. They conflate two distinct concepts in retweet prediction. The first is the dynamic, content-specific diffusion process along a cascade tree. The second is the static, global model of user–user affinity. Even when Graph Neural Networks are employed, the inherent tree structure of cascades is ignored. These models only capture single-content local relationships. They fail to encode the actual propagation mechanism that drives retweeting. Moreover, inter-user relationships, user stances toward popular topics, and global network structures remain unconsidered.

To address the above issues, this paper proposes a topical stance representation method based on topic node weighting. First, the method classifies the structure between user nodes and topic nodes according to the structural balance theory; then, it measures the importance of topic nodes and assigns weights through node features and network balance structures; finally, it reconstructs the user relationship network based on users’ stances toward topic nodes. This approach considers the information of the topic nodes in the network, effectively improving the characterization of topical stances. We present a lightweight, interpretable solution that upgrades traditional signed GNNs to a ternary “user–topic–user” perspective without heavy hyper-graph or large-model pipelines, enabling deployment on billion-edge social platforms.

In addition,this paper proposes a user retweet prediction model that integrates topical stance and graph attention network (TSGAT) method. The method first uses the user relationship network to mine user relationship and stance features; then, it employs TSGAT to extract positive and negative neighbor information of users in the retweet network; finally, it integrates positive/negative neighbor information and topical stance information to generate low dimensional representation vectors for users, enabling prediction of retweet behavior and polarity. This method effectively fuses multidimensional information such as user relationships, topical stances, and network structures, significantly improving the accuracy of user retweet behavior and polarity prediction.

This paper collects text data related to popular on social platforms, extracts topic figures and entities as topic nodes, models users’ stances toward topics as positive/negative polarity edges, and constructs a bipartite signed network containing user nodes and topic nodes. By analyzing users’ stances and attitudes toward topics, the relationship polarity between users is obtained to reconstruct the user relationship network. Then, the user relationship network is used to learn inter user relationship and stance features, and TSGAT is adopted to model the user retweet network. By fusing multidimensional user features, accurate retweet prediction is achieved. This article conducted experiments on predicting user retweet behavior and polarity on topic datasets on social media platforms such as Twitter and Weibo. The results showed that the proposed method improved the accuracy of predicting user retweet behavior and polarity.

## Related work

The prediction of user retweet behavior in social networks is a core research topic in the field of machine learning and artificial intelligence. Its core objective is to construct prediction models by analyzing users’ historical behavioral data (such as retweet frequency, interaction patterns, content preferences, etc.), combining with the structural features of social networks and the dynamics of information propagation, so as to infer the possibility of users retweet specific content in the future. This technology not only provides theoretical support for understanding the mechanism of information propagation but also demonstrates important application values in scenarios such as business decision-making, public opinion management, and personalized services. This paper classifies existing research methods into two categories: methods based on statistical analysis and methods based on graph neural networks.

Early forward prediction studies mainly relied on statistical analysis methods, which achieved prediction by modeling user features, text features, and network structures, combined with manually extracted features and probabilistic models. For example, Farshad [13] et al. used users’ behavioral features as node attributes and employed random walk methods to calculate node similarity for retweet prediction; Hong [14] et al. constructed a logistic regression model based on content features, user activity, and follower information to predict the popularity of tweets; the SEISMIC model proposed by Zhao [15] et al. modeled retweet behavior as a self-exciting point process, combining early propagators and propagation paths to predict the temporal popularity of tweets. These methods have good interpretability and computational efficiency, but due to the difficulty in obtaining node behavioral features and the uncertainty of their authenticity, they struggle to capture complex feature interactions and semantic representations, thus imposing significant limitations on prediction algorithms that rely on node features.

To preserve the temporal and topological order of information diffusion, a family of studies encodes cascades as trees rather than as flat sequences. Tai et al. (2015) introduced TreeLSTM [16] that recursively aggregates the hidden states of all children through node-specific forget gates; when the tree is a retweet path, the root representation captures the entire propagation history without losing intermediate structure. Follow-up work such as Cascade-LSTM [17] and DeepDiffuse extended TreeLSTM with top-down passes, Hawkes-style time decay, or content-aware gates, yielding strong performance in size prediction and rumour detection.

In the subfield of statistical analysis-based link prediction, Shang et al. proposed Link Prediction for Tree Networks [18], which systematically addresses link prediction in tree-structured propagation networks. To capture the structural traits of tree networks, the study introduces three interpretable static structural features: path overlap (quantifying the overlap of information propagation paths between node pairs), hierarchical distance (measuring the positional gap of nodes in the tree’s hierarchy), and subtree symmetry (evaluating the structural consistency of subtrees rooted at target nodes). By leveraging these topology-specific features, this lightweight non-neural network approach attains an approximate 95% accuracy in predicting link [19] existence in tree networks.

Notably, Shang et al.’s method centers on unsigned tree-like dynamic propagation chains, relying on static topological features to predict retweet links for specific content. In contrast, this study constructs a static signed user-user network through aggregating users’ stances across multiple topics,which is a task design distinct from dynamic content-specific propagation modeling. Our work focuses on predicting long-term user relationships and retweet polarity, where the network reconstruction hinges on dynamic topical stances rather than static topological traits of propagation chains. Most existing studies on tree-like propagation chain prediction only prioritize link existence, rarely integrating positive/negative polarity with topic semantics. This study’s innovation lies in incorporating topic stances into its modeling framework, effectively filling this critical research gap in long-term, polarity-aware retweet prediction.

In recent years, Graph Neural Networks (GNNs) have demonstrated powerful capabilities in social network retweet prediction, but existing GNN-based methods still fail to address the distinction between cascade tree dynamics and static user affinity. For instance, some studies (e.g., those relying on general graph structures to model propagation) conflate the tree-like cascade process of specific content with global user affinity networks, while others ignore the timing and path structure of cascades entirely—these oversights lead to misalignment with the actual retweet decision mechanism. Different from traditional neural network models that can only process vector or sequence data, GNNs are specifically designed to handle non-Euclidean graph data, but their application in retweet prediction has yet to fully leverage the tree-like topology of cascades, which is key to modeling how specific posts diffuse.

In 2025, Li et al. released the first benchmark that couples signed hypergraphs with heterophily-aware node features [20]. Their finding that homogeneous signed edges [21] are insufficient when neighbor labels differ directly motivates our stance-weighted butterfly structures. We follow a similar weighted-hyper-edge idea, yet TNTSR assigns topic-specific and interpretable weights instead of learning a global homophily coefficient. Also in 2025, EduLLM presented a framelet-based spectral decomposition of signed hypergraphs [22] and fused it with large language models for student-performance prediction.

Compared with these studies, we offer a lightweight and interpretable solution that upgrades traditional signed GNNs to a ternary “user–topic–user” perspective without resorting to full hyper-graph or heavy large-model pipelines, making it ready for deployment on billion-edge social platforms.

In real social scenarios, constrained by platform rules and users’ self interests, users rarely reveal polar relationships with others directly. Existing methods ignore the importance weighting of topic nodes and fail to fully utilize topic node information during relationship reconstruction, affecting the accuracy of user stance analysis, community structure identification, and retweet behavior prediction. Furthermore, existing methods lack collaborative modeling of topic node information and network structures, failing to effectively characterize the “user-topic-user” ternary relationship; the inefficiency of multidimensional feature fusion mechanisms makes it difficult to process multisource heterogeneous information such as user features, topic semantics, and network edge polarities simultaneously, thus restricting the generalization ability of models in complex retweet scenarios. These issues hinder the prediction of user retweet behavior and polarity, which urgently requires more effective solutions.

## Preliminary

### Symbolic networks and binary networks

In real social networks, nodes usually represent users and have inherent attributes that reflect personal information, preferences, or positions. The edges between nodes can represent relationships such as following. In the task of predicting user retweet, existing research has shown that edges are mostly unipolar, indicating only the existence of retweet behavior. However, in this paper, positive and negative polarities are added to edges to reflect users’ attitudes towards retweet text. This type of network is called a symbolic network [23]. To obtain the implicit polarity of retweeting relationships between users, this paper introduces topic nodes representing popular public opinion topics and related entities. At this time, edges only exist between users and topic nodes, reflecting users’ positions on the topic. This type of network, which includes two types of nodes, edges only exist between heterogeneous nodes, and has positive and negative polarity, is called a binary symbol network.

**Fig 1:**
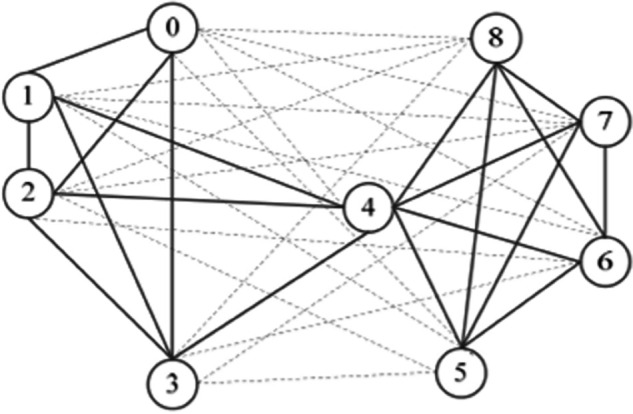
Voting network for US supreme court justices.

A signed network can be described as $G=\left(V,E_{+},E_{-}\right)$, where the node set is $V=\left(v_{1},v_{2},\cdots ,v_{n}\right)$, the positive edge set is $E_{+}=\left\{E_{ij}^{+}|v_{i},v_{j}\in V\right\}$, and $E_{ij}^{+}$ represents a positive edge between nodes $v_{i}$ and $v_{j}$, denoting positive relationships such as approval, friendship, or trust. The negative edge set is $E_{-}=\left\{E_{ij}^{-}|v_{i},v_{j}\in V\right\}$, where $E_{ij}^{-}$ represents a negative edge between nodes *v*_*i*_ and $v_{j}$, denoting negative relationships such as opposition, enmity, or distrust. An example of a signed network is shown in Fig 1, which describes the voting behavior of 9 justices of the U.S. Supreme Court in 2007.

**Fig 2:**
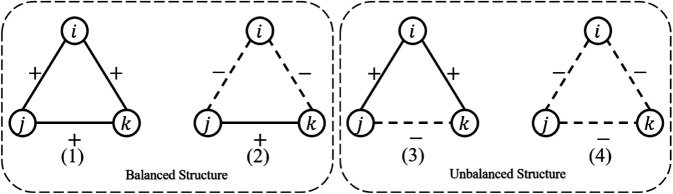
The triangular structure of symbolic networks.

The theory of structural balance is the theoretical foundation of symbolic network research, providing a basis for the structural analysis of symbolic networks. The structural balance theory in signed networks is mainly discussed based on triangular balanced structures, which consist of three nodes and three edges. Each edge can be positive or negative, resulting in four combinations. These combinations form four intuitive understandings: (1) a friend of a friend is a friend; (2) an enemy of a friend is an enemy; (3) a friend of an enemy is an enemy; (4) an enemy of an enemy is a friend. As shown in Fig 2, structures (1,2) are balanced, while (3,4) are unbalanced. Studies in Reference [24] have shown that the number of balanced structures in real networks is much larger than unbalanced ones, and unbalanced structures tend to transform into balanced ones as the network evolves.

Edges can be represented by an adjacency matrix *A*, where *A*_*ij*_ represents the relationship between node *u*_*i*_ and $v_{j}$: *A*_*ij*_ = 1 for a positive edge, *A*_*ij*_ = 0 for no edge, and *A*_*ij*_ = −1 for a negative edge.

**Fig 3:**
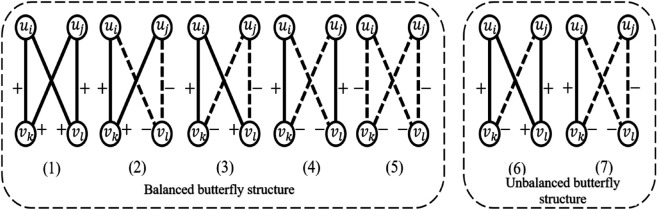
The butterfly structure of binary symbolic networks.

In classical bipartite networks, a common research structure is the butterfly structure [25], which provides the most natural framework for studying bipartite signed networks [26]. Fig 3 shows seven types of signed butterfly structures, among which five are balanced and two are unbalanced according to the structural balance theory.

This paper uses the notation (*,*,*,*) to represent a signed butterfly structure, denoting the edge cycle between two node types. The simplest types are (+,+,+,+) and (–,–,–,–), as shown in Fig 3(1) and (5), which represent all positive or all negative edge structures. Both are balanced due to the even number of negative edges. The (+,+,+,+) structure indicates that two users hold positive stances toward both topics. The (+,+,+,−) and (+,−,−,−) structures are unbalanced with an odd number of negative edges. The butterfly structure in Fig 3(6) includes all cases with a single negative edge, where two users always share the same stance on one topic but differ on the other, leading to conflicts. The (+,−,−,−) structure in Fig 3(7) can be seen as the complementary form of (+,+,+,−), exhibiting similar contradictions. The (+,+,−,−) structure in Fig 3(2) shows that two users share positive stances on one topic and negative stances on another, reflecting similar attitudes. The (+,−,+,−) structure in Fig 3(3) indicates that one user holds positive stances on both topics while the other holds negative stances, reflecting opposite attitudes. The (+,−,−,+) structure in Fig 3(4) shows that both users hold positive stances on one topic and negative stances on the other, but their attitudes are completely reversed.

### Construction of user topic network

To study the stance distribution of social network users in popular topics and predict users’ retweet behaviors, this paper collects relevant data on the following six popular topics from real social platforms such as Twitter and Weibo: the 2024 Taiwan regional election event, the 2024 U.S. presidential election event, the Israeli Palestinian conflict event, the death of the founder of Wahaha, the Mayday lip-syncing controversy, and the Joker Xue screen recording controversy. Data collection was performed through publicly available APIs in accordance with Twitter’s Terms of Service and Weibo’s Platform Rules. No personal identifiers were collected or stored. Data collection was performed through publicly available APIs in accordance with Twitter’s Terms of Service and Weibo’s Platform Rules. No personal identifiers were collected or stored.

**Fig 4:**
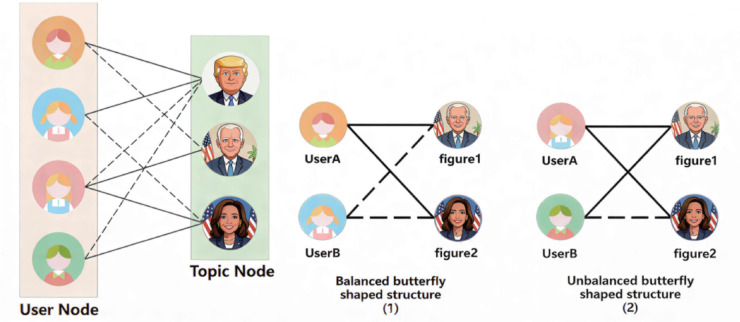
US presidential election. A: User topic network. B: The butterfly structure of social networks. The political caricatures were generated by the authors using Google Gemini AI. All other elements were created by the authors.

This article extracts people or entities related to different hot topics from user text data as topic nodes. For example, on the topic of the US presidential election, Trump and Harris who participated in the election were extracted as topic nodes, as shown in Fig 4. Since there are extractable topic tags in the text of the microblog platform, this paper also extracts relevant sub topic tags as topic nodes from the text of microblog topics such as the death of Wahaha’s founder, the controversy of Mayday’s lip synching, and the controversy of Jacky Xue’s screen photography. The specific topic data is shown in Table 1.

**Table 1:** Dataset overview extracted from social-media hot topics.

Topic	Period	Number of texts	Brief description
Taiwan elections	2022.10–2024.01	413 148	Three-party race (KMT, DPP, TPP) in 2024 Taiwan election; DPP candidate won.
US Presidential election	2024.03–2024.11	476 072	2024 US presidential election; Republican candidate Trump won.
Israeli–Palestinian conflict	2023.10–2023.12	722 342	Oct-2023 attack on Israel by Hamas.
Wahaha	2024.02–2024.04	477 835	Death of Wahaha founder Zong Qing-hou; old feud with Nongfu Spring founder revisited by self-media.
Mayday lip-synch	2023.12–2024.01	272 370	Famous band Mayday accused of lip-synching at concerts.
Jacky Xue screen-shot	2024.02–2024.03	147 497	Singer Jacky Xue posted cinema-screen photos in his movie review, causing piracy controversy.

For each hot topic, we collect topic related posts, platform supplied keywords, and trending titles as raw data. Keywords and named entity recognition are then applied to extract persons, entities, and sub-topics. After counting their mention frequencies, those with high frequency and substantial discussion are retained as topic nodes.

Taking the 2024 U.S. election on Twitter as an example, we extract entities such as parties, candidates, and senators e.g., Republican & Democratic parties and their nominees Trump and Harris. On Weibo, besides post text, we also mine sub-topics from trending hashtags. For topics such as the death of Wahaha founder, Mayday lip-synch dispute, and Jacky Xue’s cinema-recording controversy, representative hashtags include #Zong Qinghou passed away#, #Nongfu Spring’s 9-year-old employees speak out#, #Mayday lip syncing appraisal#, and #Reflections on Flying Life 2#, which are selected as topic nodes.

After node extraction, sentiment analysis is performed on user text to identify polarities toward each topic node. Positive and negative sentiments are modeled as positive and negative edges, yielding a user–topic bipartite network (an example is shown in Fig 4).

After building a user topic network, this article reconstructs the relationships between users based on their emotional polarity towards the same topic. Due to the fact that the edges of a binary symbolic network only exist between nodes of different types, based on the theory of structural balance, a third edge between two user nodes is constructed based on the two edges between them and their common neighbors, resulting in a symbolic network composed of user nodes. When nodes *i* and *j* in user node set *U* have edges with the same sign as node *k* in topic node set *V*, a positive edge is constructed between them. When nodes *i* and *j* have edges with different signs, a negative edge is constructed between them. Consider all common neighbors of nodes *i* and *j*, and determine the final symbol based on the symbol ratio of all connected edges.

The above construction method treats all topic nodes equally, masking their differences. For example, in the US election, the discussion level of Trump and his deputies is much higher than that of Democratic candidates, and Musk, who supports Trump, also has a higher discussion level but lower than other candidates. This leads to the distortion of directly constructed user relationships by ignoring the differences in discussion, attention, and controversy among topic nodes. Therefore, this article distinguishes topic nodes based on heterogeneity and assigns greater weight to nodes with high topic and controversy to ensure the accuracy of user relationship construction.For topic nodes with high discussion and controversy, they should be given greater weight because the polarity of relationships between users mainly stems from differences in their positions towards these hot topics, while peripheral characters with low discussion are relatively less concerned in the topic and have less impact on user relationships. In summary, this article differentiates the topic node set through heterogeneity and assigns greater weights to nodes with greater topicality and controversy to ensure the accuracy of user relationship construction.

In order to measure the degree of discussion and controversy of topic nodes in the topic, and to differentiate the heterogeneity of the topic node set, this paper first analyzes the node characteristics. The discussion level of the topic character is determined by the number of mentions in the user’s text, so the size of the topic node degree can intuitively reflect the user’s discussion and attention to it. In binary symbolic networks, topic nodes have two types of edges, positive and negative. Therefore, the positive and negative degrees of nodes can respectively represent the number of users who support and oppose them, which to some extent reflects the controversial nature of topic nodes among users. In addition, topic nodes with mostly positive or negative degrees tend to cluster neighboring users into a tight group, lacking differentiation of differences in positions between users. On the other hand, topic nodes with similar degrees can form two opposing groups of positive and negative neighbors, supporting and opposing each other. This not only gathers users with similar positions, but also divides users with different positions, making the representation of topic positions more effective.

Additionally,This article analyzes the local structure of the network, and in binary symbolic networks, the butterfly structure is the key to studying structural equilibrium theory. In social networks, a balanced structure can clearly divide nodes, as shown in Fig 4. Although an unbalanced structure can lead to unstable topic positions, it reflects subtle differences between users with the same position and similarities between users with different positions. Compared with the positive and negative values that reflect the controversial nature of the topic nodes among all users, the unbalanced structure further reflects the controversial nature of the topic nodes among users with the same stance.

Traditional structural balance methods treat every topic node as equally influential. They ignore discussion volume, controversy, and stance discriminative power. This distorts user relationship inference. They count balanced or unbalanced butterflies to validate consistency. Yet they fail to exploit these structures for finer stance distinctions.

TNTSR addresses the limitations of traditional methods through targeted innovations aligned with structural balance theory and network representation learning: it introduces a heterogeneous weighting mechanism for topic nodes, integrating each topic’s controversy and stance discriminability to assign weights that ensure influential topics dominate user relationship inference, replacing traditional binary relationship polarity with a continuous measure of stance alignment strength; it incorporates a stance membership matrix to model global user communities, optimizing a joint objective that balances local neighbor similarity and global community separation, ensuring users with similar stances cluster densely while those from different groups are spatially separated in embedding space; and by preserving detailed topic node information and capturing global stance structures, it generates more discriminative user embeddings that better link user stances to retweet behavior, laying a stronger theoretical and practical foundation for downstream retweet prediction models like TSGAT.

## Topical stance representation

### Problem definition and overall framework

**Fig 5:**
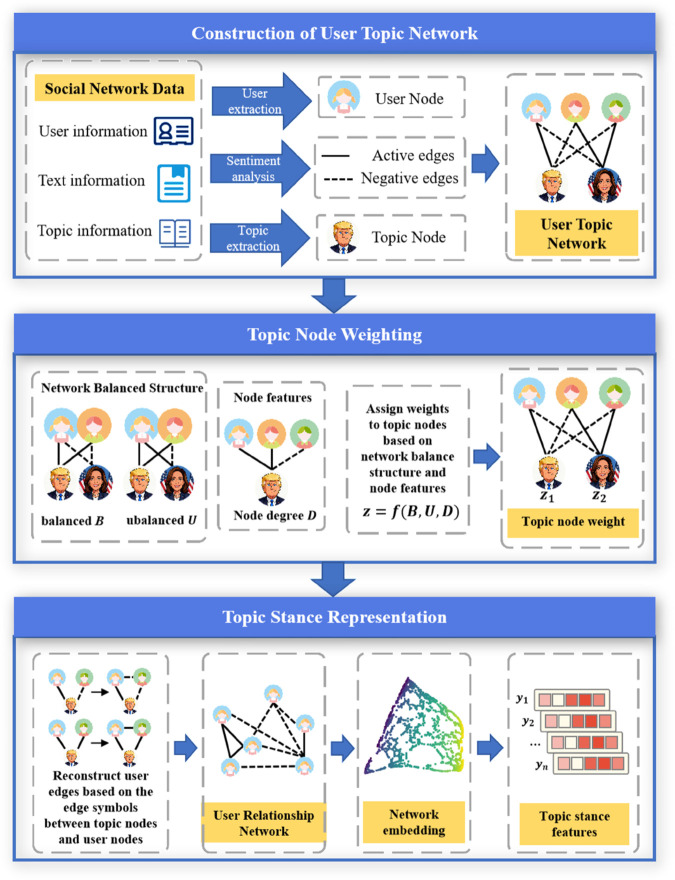
Overall framework of topical stance representation method based on topic node weighting. The political caricatures contained within this figure were generated by the authors using Google Gemini AI. All other elements were created by the authors.

This section mainly introduces the modeling method of user topic network and the topical stance representation method, first giving the problem definition. Consider a bipartite signed network $G=\left(U,V,{ℰ}_{+},{ℰ}_{-}\right)$ modeled based on social network. Nodes in *U* represent users in social networks, nodes in *V* represent popular topics or related figures in social networks. Edges describe the relationships between nodes and can also be represented by an adjacency matrix $A\in R^{n_{U}\times n_{V}}$. Based on the above problem definition and discussion, this paper proposes a Topic Node weighting based Topical Stance Representation (TNTSR) method, as shown in Fig 5.

### Topical stance representation method

This article comprehensively measures the discussion and controversy of topic nodes in the topic based on node degree, node positive and negative degree ratio, and unbalanced structure ratio, and weights the heterogeneity of nodes. The weight of node k can be expressed as Eq (1)

$$z_{k}=\left[1-{\left(2\cdot \frac{d_{k}^{+}}{d_{k}^{+}+d_{k}^{-}}-1\right)}^{2}\right]+\frac{U_{k}}{U_{k}+B_{k}},$$ (1)

Among them, $d_{k}^{+}$ represents the positivity of a node, $d_{k}^{-}$ represents the negative degree of a node. *U*_*k*_ represents the number of unbalanced structures composed of node *k* and its neighbors, while *B*_*k*_ represents the number of balanced structures composed of node *k* and its neighbors.

$$w_{ij}=\frac{\sum_{k\in V}A_{ik}A_{jk}z_{k}}{\sum_{k\in V}|A_{ik}A_{jk}z_{k}|},$$ (2)

Among them, *A*_*jk*_ represents the edge symbol between node *i* and node *k*, and *A*_*jk*_ represents the edge symbol between node *j* and node *k*.

After constructing the user relationship network, the adjacency matrix corresponding to the user nodes can be obtained. This article refers to the idea of Laplace feature mapping, where the weights of the adjacency matrix represent the degree of similarity between the positions of two user nodes on popular topics. If the similarity between two nodes is higher, their low dimensional spatial embeddings should be closer. Therefore, the objective function can be obtained as follows:

$$\min_{Y}\frac{1}{2}\sum_{i=1}^{n}\sum_{j=1}^{n}\|y_{i}-y_{j}{\|}_{2}^{2}W_{ij}.$$ (3)

Among them, *y*_*i*_ represents the embedding vector of node *i*, and $Y=\left(y_{1},\cdots ,y_{n}\right)$ is the local embedding matrix of the node.

However, the above method only considers the local neighbor information of nodes and ignores the network structure information. In social networks, users gather and oppose each other due to different positions on topics, forming multiple groups. In order to highlight the aggregation and opposition of groups in embedding, and thus express the topical stance, this paper introduces the community membership degree of nodes and modifies it on the basis of traditional community membership degree to obtain targeted node stance membership degree to represent the user’s membership group in the network.

The community structure of the network is defined as: within the community, nodes are relatively tightly connected, while the connections between the community and external communities are relatively sparse. Generally, in terms of structure, communities are relatively independent, with each node belonging to only one community. Community information is the label of the community that represents that the node belongs to a certain community in the network. The position membership value in this article is not a strict membership relationship of nonzero or one, but any value between 0 and 1. A node can belong to different positions with different degrees of membership. The network position membership matrix is represented as $C=c_{ui}^{l}\;\times \;n$, where *c*_*ui*_ represents the degree of membership between position *u* and node *i*, *n* is the number of nodes, *l* is the membership dimension, and has the following constraints

$$\sum_{u=1}^{l}c_{ui}=1.$$ (4)

According to the above definition of the degree of community membership, each column of the matrix represents the membership relationship between a node and all positions, and the position with the highest degree of membership is usually used to represent the node position label.

Considering node positions in the process of network embedding not only promotes the optimization of the embedding of the nodes, but also makes the distribution of nodes with the same position denser, while the distribution of nodes with different positions is relatively sparse, making the results of network representation more distinguishable and effective, and making the results of the embedding of the node position more accurate.

Using the position membership matrix to adjust the network adjacency matrix, the more similar the membership between nodes, the greater the weight of the edges between nodes. Then, based on the adjusted adjacency matrix, the node embedding matrix is reoptimized to form a cyclic iteration. In summary, the following convex optimization problem can be established:

$$\min_{Y,C}\frac{1}{2}\sum_{i=1}^{n}\sum_{j=1}^{n}{||y_{i}-y_{j}||}_{2}^{2}(aW_{ij}-{||c_{i}-c_{j}||}_{2}^{2})\;\text{s.t.}\;Y^{T}DY=I,\;C^{T}1_{l\times n}=1_{n\times n}.$$ (5)

The matrix *D* is the degree matrix of the network. α>0 is a parameter used to balance the impact of errors between network edge weights and node membership degrees.

In the above optimization problem, there are two constraints. The first is the constraint on the embedding scale of nodes, which ensures that the nodes are evenly distributed in space. The second is the constraint on the degree of membership of the nodes, which ensures that the sum of all degrees of membership of each node is 1.

## Topical stance and graph sttention network

### Overall framework of the model

**Fig 6:**
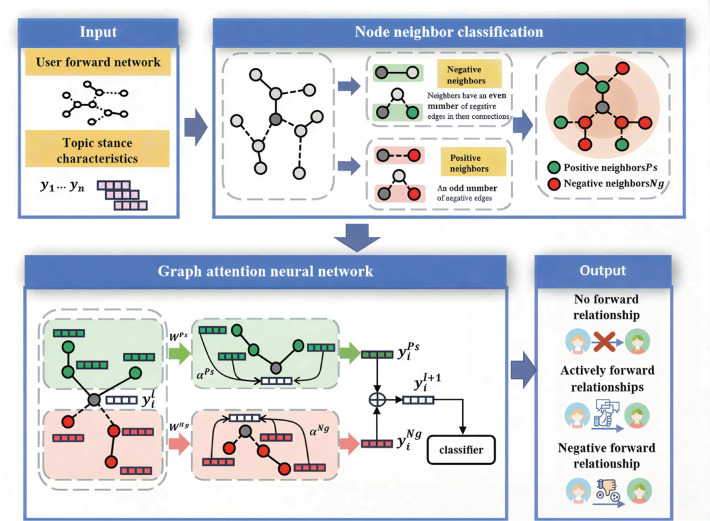
Overall framework of graph attention neural network user retweet prediction method that integrates topical stance.

This paper proposes a user retweet prediction method that integrates Topic Stance and Graph Attention neural network (TSGAT), as shown in Fig 6. First, the positive and negative neighbors of nodes are divided according to the edge polarity of the user retweet network. Then, the topic stance features are used as the initial input, and the Graph Attention neural network is used to learn the information of the positive and negative neighbors of users in the network respectively. Finally, the neighbor information of users and the network stance information are comprehensively considered and fused to obtain the low-dimensional representation vector of users, so as to predict the retweet behavior and polarity. The rest of this chapter will introduce each part of the TSGAT method in detail.

### Graph attention neural network model integrating topical stance

Consider a symbolic network $G=\left(U,\varepsilon_{+},\varepsilon_{-}\right)$ modeled based on user retweet networks, where $U=\left\{u_{1},u_{2},\cdots ,u_{n}\right\}$ represents a set of n nodes, where nodes represent users in a social network. $\varepsilon_{+}\subset U\times U$ and $\varepsilon_{-}\subset U\times U$ represent sets of positive and negative edges, respectively, representing positive and negative retweet relationships between users. Note that $\varepsilon_{+}\cap \varepsilon_{-}=\emptyset$ means a pair of nodes cannot have both positive and negative edges at the same time. The adjacency matrix of symbol network *G* can be represented by $A\in R^{n\times n}$, where *A*_*ij*_ = 1 represents the existence of a connection between node *u*_*i*_ and node *u*_*j*_ on a positively connected edge, *A*_*ij*_ = −1 represents a negatively connected edge, and if *A*_*ij*_ = 0, it indicates that there is no connection between node *u*_*i*_ and node *u*_*j*_. With the above symbols and definitions, the problem of predicting user retweet can be formally defined:

In a user retweet network, based on existing user retweet information in the network, predict the possibility of a retweet relationship between two unrelated users, as well as whether the polarity of the retweet relationship is positive or negative. Specifically, given a user retweet network $G=\left(U,\varepsilon_{+},\varepsilon_{-}\right)$, the goal is to learn a function that predicts the likelihood of edge connections between nodes that have not yet formed edges based on existing edge information. This function maps each pair of nodes $\left(u_{i},u_{j}\right)$ to a triplet $\left(P_{ij}^{+},P_{ij}^{-},P_{ij}^{\emptyset }\right)$, where $P_{ij}^{+}$ represents the probability of positive edges between node *u*_*i*_ and node *u*_*j*_, $P_{ij}^{-}$ represents the probability of negative edges between node *u*_*i*_ and node *u*_*j*_, and $P_{ij}^{\emptyset }$ represents the probability of no edges between node *u*_*i*_ and node *u*_*j*_,$P_{ij}^{+}+P_{ij}^{-}+P_{ij}^{\emptyset }=1$.

Based on the above problem definition and discussion, this paper proposes a user retweet prediction model that integrates topical stances.

The GAT used in this article is a graph neural network model based on attention mechanism. Compared with the traditional neighbor node aggregation method, GAT adopts a more efficient attention mechanism to aggregate the information of neighboring nodes. This attention mechanism allows GAT to learn the different contributions of neighboring nodes under different tasks, better understanding the complex relationships between nodes.

This article is based on a user topic network, which reconstructs the relationships between users to represent their stance on popular topics. The embedding results are input into the GAT model as a set of user features $y=\left\{y_{1},y_{2},\cdots ,y_{n}\right\}$. After passing through the graph attention layer, the input user node features y are linearly transformed into a new feature sequence $y^{\prime }$. Through this learnable transformation, the attribute features of neighboring nodes in the user retweet network are captured, and the impact of each retweet neighboring node on the current user node is calculated, thereby transforming the input features into more reasonable feature representations.

Traditional GAT models may treat negative edges in symbolic networks as negations of positive ones. simply treating negative edges as negation of positive edges is an incorrect assumption, as negative edges have different definitions and semantically represent different meanings in real networks [27]. Based on the theory of symbolic society, there are complex associations between positive and negative edges. If they can be extracted, they can provide richer network information. Therefore, the following will discuss the theory of structural balance in symbolic networks and utilize it to simultaneously capture both positive and negative edges during the aggregation process.

**Fig 7:**
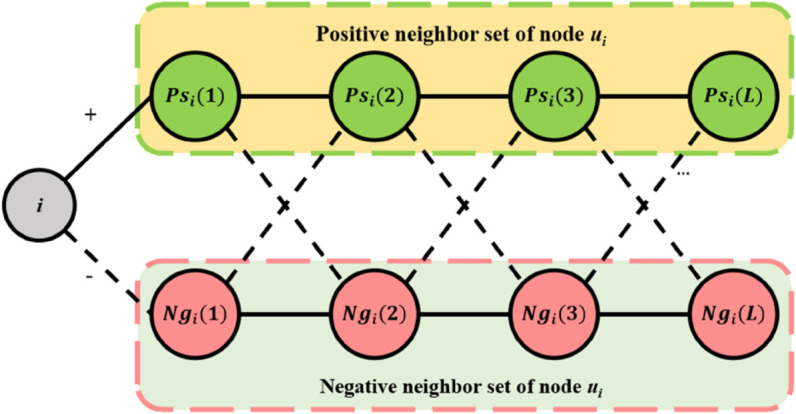
Classification of positive and negative neighbor sets.

In the Preliminary section of the article, the structural balance theory of symbolic networks has been introduced, which classifies triangular structures in symbolic networks as balanced or unbalanced. Structures containing an even number of negative edges are considered balanced, while structures containing an odd number of negative edges are considered unbalanced. Based on these definitions, it can be seen that if there is a path of length *l* and containing an even number of negative edges between node *u*_*i*_and node *u*_*j*_, then structural balance theory implies the existence of positive edges between node *u*_*i*_ and node *u*_*j*_. As shown in Fig 7, starting from node *u*_*i*_, it demonstrates how all symbolic edges of a given length allocate the endpoint node to the positive and negative sets of node *u*_*i*_.

Among them, $N_{i}^{+}$ is defined as the positive edge neighbor set of node *u*_*i*_, that is, if *A*_*ij*_ = 1, then $u_{j}\in N_{i}^{-}$. Let the negative edge neighbor set of node *u*_*i*_ be denoted as $N_{i}^{-}$, where when *A*_*ij*_ = −1, $u_{j}\in N_{i}^{-}$. In Fig 7, it can be seen that if there is a balanced path of length *l* (i.e. $u_{k}\in P_{s_{i}}(l)$) between node *u*_*k*_ and node *u*_*i*_, all positive edge neighbors of node *u*_*k*_ will be placed in the positive set $P_{s_{i}}(l\;+\;1)$. This is because adding a directly connected edge to a balanced path still results in a balanced path, but with an increase in length. On the contrary, adding a negative edge to a balanced path will result in an unbalanced path. All negative neighbors of node *u*_*k*_ will be placed in the negative set $N_{g_{i}}(l\;+\;1)$. Based on the above analysis, the recursive definition for calculating positive and negative sets from the perspective of node *u*_*i*_ is as follows:

when *l* = 1,

$$P_{s_{i}}(1)=\left\{u_{j}|u_{j}\in N_{i}^{+}\right\},$$ (6)

$$N_{g_{i}}(1)=\left\{u_{j}|u_{j}\in N_{i}^{-}\right\}.$$ (7)

When *l* > 1,

$$P_{s_{i}}(l+1)=\left\{u_{j}|u_{k}\in P_{s_{i}}(l)\text{ and }u_{j}\in N_{k}^{+}\right\}\;\;\cup \left\{u_{j}|u_{k}\in N_{g_{i}}(l)\text{ and }u_{j}\in N_{k}^{-}\right\},$$ (8)

$$N_{g_{i}}(l+1)=\left\{u_{j}|u_{k}\in N_{g_{i}}(l)\text{ and }u_{j}\in N_{k}^{+}\right\}\;\;\cup \left\{u_{j}|u_{k}\in P_{s_{i}}(l)\text{ and }u_{j}\in N_{k}^{-}\right\}.$$ (9)

**Fig 8:**
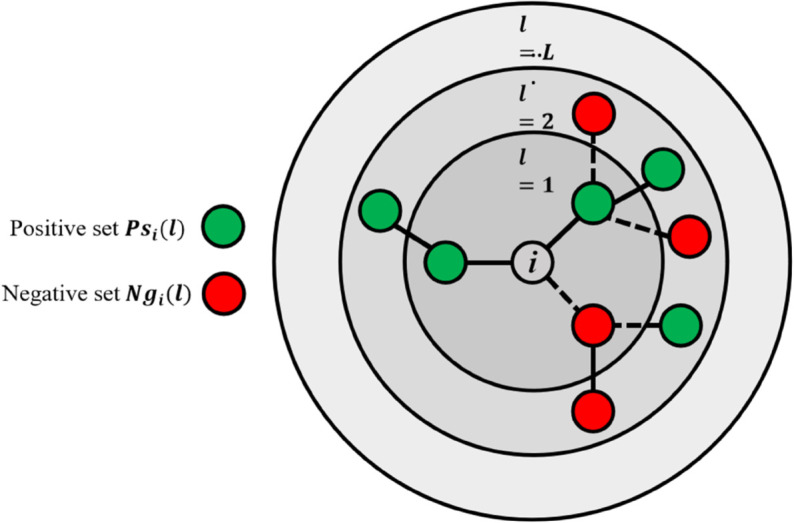
Node neighbor information aggregation mechanism.

Fig 8 illustrates how to aggregate neighbor information through attention mechanisms in a symbolic network. Among them, the circles marked as l=1,2,⋯,L are used to represent how far the node is from the starting point *u*_*i*_, and also indicate which hierarchical node information will be aggregated into the learning representation of node *u*_*i*_in the TSGAT model of this paper. In the figure, it can be observed that there are separate attention mechanisms responsible for integrating information from both positive and negative sets. For example, in the first layer of the graph, the two positive edge neighbors of node *u*_*i*_will be incorporated into the first level positive representation through attention mechanism a.

When aggregating information in the TSGAT model of this article, two representations will be maintained for each layer, one corresponding to the positive set of nodes and the other corresponding to the negative set. Similar to unsigned GAT, use $y_{i}^{\left(0\right)}\in {\mathbb{R}}^{d_{in}}$ to represent the initial features of node *u*_*i*_. Therefore, for the lth aggregation layer, use the following method to calculate the attention mechanism:

$$e_{ij}^{P_{s}(l)}=\text{LeakyReLU}\left(a^{T}\left[Wy_{i}^{\left(l\right)}∥Wy_{j}^{\left(l\right)}\right]\right),\;u_{j}\in N_{i}^{+},$$ (10)

$$e_{ik}^{N_{g}(l)}=\text{LeakyReLU}\left(a^{T}\left[Wy_{i}^{\left(l\right)}∥Wy_{k}^{\left(l\right)}\right]\right),\;u_{k}\in N_{i}^{-},$$ (11)

Normalized to obtain:

$$\alpha_{ij}^{P_{s}(l)}=\frac{\exp\left(e_{ij}^{P_{s}(l)}\right)}{\sum_{j^{\prime }\in N_{i}^{+}}\exp\left(e_{ij^{\prime }}^{P_{s}(l)}\right)},$$ (12)

$$\alpha_{ik}^{N_{g}(l)}=\frac{\exp\left(e_{ik}^{N_{g}(l)}\right)}{\sum_{k^{\prime }\in N_{i}^{-}}\exp\left(e_{ik^{\prime }}^{N_{g}(l)}\right)},$$ (13)

In the first aggregation layer, calculate the aggregation using the following method:

$$y_{i}^{P_{s}(1)}=\sigma \left(W^{P_{s}(1)}\left[\sum_{j\in N_{i}^{+}}\alpha_{ij}^{P_{s}(0)}y_{j}^{\left(0\right)},y_{i}^{\left(0\right)}\right]\right),$$ (14)

$$y_{i}^{N_{g}(1)}=\sigma \left(W^{N_{g}(1)}\left[\sum_{k\in N_{i}^{-}}\alpha_{ik}^{N_{g}(0)}y_{k}^{\left(0\right)},y_{i}^{\left(0\right)}\right]\right),$$ (15)

Among them, σ() is a nonlinear activation function, $W^{P_{s}(l)}$, $W^{N_{g}(l)}\in {\mathbb{R}}^{d_{out}\times 2d_{in}}$ is a linear transformation matrix responsible for obtaining positive and negative representations from sets $P_{s_{i}}\left(l\right)$ and $N_{g_{i}}\left(l\right)$, and *dout* is the length of the two internal hidden representations.

Aggregation is more complex in all subsequent layers. The definition of aggregation is as follows When *l* > 1:

$$y_{i}^{P_{s}(l)}=\sigma \left(W^{P_{s}(l)}\left[\sum_{j\in N_{i}^{+}}\alpha_{ij}^{P_{s}(l-1)}y_{j}^{P_{s}(l-1)},\sum_{k\in N_{i}^{-}}\alpha_{ik}^{N_{g}(l-1)}y_{k}^{N_{g}(l-1)},y_{i}^{P_{s}(l-1)}\right]\right),$$ (16)

$$y_{i}^{N_{g}(l)}=\sigma \left(W^{N_{g}(l)}\left[\sum_{j\in N_{i}^{+}}\alpha_{ij}^{P_{s}(l-1)}y_{j}^{N_{g}(l-1)},\sum_{k\in N_{i}^{-}}\alpha_{ik}^{N_{g}(l-1)}y_{k}^{P_{s}(l-1)},y_{i}^{N_{g}(l-1)}\right]\right).$$ (17)

Among them $W^{P_{s}(l)},W^{N_{g}(l)}\in {\mathbb{R}}^{d_{out}\times 3d_{out}}$. When collecting the positive representation of node *u*_*i*_in layer *l* > 1, i.e. $y_{i}^{Ps\left(l\right)}$, it is the aggregation of the positive representation information $y_{i}^{P_{s}(l-1)}$ of all positive edge neighbors $u_{j}\in N_{i}^{+}$ in layer *l*–1, as well as the negative representation information $y_{k}^{Ng\left(l-1\right)}$ of all negative edge neighbors $u_{k}\in N_{i}^{-}$, under the attention mechanism. Therefore, in *l* = 2, it can be seen that positive representation not only collects information from direct positive neighbors, but also from friends of friends and enemies of enemies at the two hop level. Meanwhile, in *l* = 2, the negative representation $y_{i}^{Ng\left(2\right)}$ of node *u*_*i*_ includes the direct negative edge neighbor information collected in the first layer, as well as the enemies of node *u*_*i*_’s friends and their friends collected in the second layer.

Compared with the traditional GAT model, TSGAT has to handle both positive and negative edges. Therefore, the input features of each node are mapped into a positive representation and a negative representation by two separate linear transformation matrices; moreover, the attention coefficients for positive and negative edges have to be computed independently. Consequently, the time complexity of one TSGAT layer is twice that of a standard GAT layer, i.e.,$𝒪(2|V|\cdot 2d_{\text{in}}\cdot d_{\text{out}}+2|E|\cdot d_{\text{in}})$, where $𝒪(2|V|\cdot 2d_{\text{in}}\cdot d_{\text{out}})$ accounts for the feature linear transformation of all nodes, and $𝒪(2|E|\cdot d_{\text{in}})$ accounts for the attention-score computation. When the layer index *l* > 1, the complexity becomes$𝒪(2|V|\cdot 3d_{\text{out}}\cdot d_{\text{out}}+2|E|\cdot 2d_{\text{out}})$. Hence, the overall time complexity of TSGAT can be simplified to

$$𝒪(L(|V|\cdot d_{\text{out}}+|E|)\cdot d_{\text{out}}),$$ (18)

which shows that the numbers of nodes and edges are the main factors affecting computational efficiency.

This paper designs an objective function for learning the parameters of TSGAT. This objective function consists of two parts, both aiming to make the node representation vectors reflect the edge relationship between node pairs.

The first part introduces an additional layer, which is used as a weighted multiple logistic regression classifier. The role of this classifier is to classify node embeddings and determine whether they belong to a positive edge, a negative edge, or no edge. Specifically, construct a small batch set of nodes and then a set *M* containing triples $\left(u_{i},u_{j},s\right)$. This triple represents the node pair $\left(u_{i},u_{j}\right)$ and s∈{+,−,⊘} is used to indicate whether there is a positive edge, a negative edge, or no edge between them. Use the embedded connection $\left[z_{i},z_{j}\right]$ of node *u*_*i*_ and node *u*_*j*_ as the input of the classifier. $\omega_{s}$ represents the weight related to class *s*.

Then introduce the second part. This part is based on the extended structural balance theory and is controlled by λ to balance the contribution to the overall objective. The goal of the second part is to make nodes with positive edges in the low dimensional embedding space closer than nodes with no edges, and nodes with no edges should be closer than nodes with negative edges. The overall objective is formulated as follows:

$$ℒ(\theta^{W},\theta^{C})=-\frac{1}{\left|ℳ\right|}\sum_{(u_{i},u_{j},s)\in ℳ}\omega_{s}\log\frac{\exp\left(\left[z_{i},z_{j}\right]\theta_{s}^{C}\right)}{\sum_{q\in \{+,-,⊘\}}\exp\left(\left[z_{i},z_{j}\right]\theta_{q}^{C}\right)}\;\;+\lambda [\frac{1}{\left|{ℳ}_{\left(+,⊘\right)}\right|}\sum_{(u_{i},u_{j},u_{k})\in {ℳ}_{\left(+,⊘\right)}}\max\left(0,\left(\|z_{i}-z_{j}{\|}_{2}^{2}-\|z_{i}-z_{k}{\|}_{2}^{2}\right)\right)\;\;+\frac{1}{\left|{ℳ}_{\left(-,⊘\right)}\right|}\sum_{(u_{i},u_{j},u_{k})\in {ℳ}_{\left(-,⊘\right)}}\max\left(0,\left(\|z_{i}-z_{k}{\|}_{2}^{2}-\|z_{i}-z_{j}{\|}_{2}^{2}\right)\right)]\;\;+\text{Reg}(\theta^{W},\theta^{C}),$$ (19)

Among them, $\theta^{W}$ represents the weight matrix used in the TSGAT layer, and $\theta^{C}$ represents the parameters of the classifier. $\omega_{s}$ is used for the weight associated with class *s* (where s∈{+,−,⊘} corresponds to the categories of positive edges, negative edges, and no - edge categories). ${ℳ}_{\left(+,⊘\right)}$ and ${ℳ}_{\left(-,⊘\right)}$ are respectively the sets of node pairs with positive edges and negative edges. In each edge pair $\left(u_{i},u_{j}\right)$, another node *u*_*k*_ is further randomly sampled (and different in each period), and this node has no edge with *u*_*i*_. $\text{Reg}(\theta^{W},\theta^{C})$ is the regularization term used on the model parameters [28].

## Results

### Dataset

In this section, the effectiveness of the TSGAT model proposed in this paper will be verified through experiments. First, the specific configuration of the experiments in this chapter and the adopted datasets will be introduced. Then, the user reposting behavior prediction experiment will be used to predict whether reposting behavior occurs between users, and the user reposting polarity prediction experiment will be used to predict the positive or negative polarity of reposting emotions between users, so as to test and evaluate the TSGAT model.

This paper uses the six social network datasets introduced in the Construction of user topic network section to conduct experiments on user reposting behavior prediction and user reposting polarity prediction. The reposting relationships and reposting emotions between users are extracted from the data to construct a signed user reposting network. The specific information of the datasets is shown in Table 2.

**Table 2:** User retweet prediction experimental dataset.

Data set	Node number	Edge number	Positive edge ratio	Negative edge ratio
Taiwan elections	13510	68854	0.794	0.206
US Presidential election	10161	79917	0.689	0.311
Israeli Palestinian conflict	12515	74378	0.545	0.455
Wahaha	10968	81575	0.513	0.487
Mayday lip-synch	7605	62337	0.572	0.428
Screen shot by Jacky Xue	1981	4431	0.671	0.329

This paper uses the F1 value and the AUC value to evaluate the user reposting prediction results of the model on different datasets. F1 (F1 Score) is an indicator for evaluating the precision of a binary classification model. It takes into account both the precision and recall of the classification model simultaneously and can be regarded as a harmonic mean of the model’s precision and recall, with a range between 0 and 1.

$$F1=2\times \frac{\text{Precision}\cdot \text{Recall}}{\text{Precision}+\text{Recall}},$$ (20)

AUC (Area Under the ROC Curve) is a commonly used indicator for evaluating the performance of a classification model and is usually used to measure the accuracy of a model in binary classification problems. The ROC curve is the abbreviation of Receiver Operating Characteristic Curve, which describes the relationship between the True Positive Rate and the False Positive Rate under different classification thresholds. The AUC represents the area under the ROC curve, with a range between 0 and 1.

### Comparison methods

The method in this paper is compared with several baselines:

- SGCN [29]: GCN extends GCN to signed networks with a message-passing mechanism. Using structural balance theory, it aggregates info from positive and negative neighbors to form temporary node representations.
- SiGAT [30]: SiGAT extends GAT to signed networks by integrating network signatures, based on structural balance and status theories.
- SDGNN [31]: SDGNN leverages sociology’s structural balance theory to understand signed network node relationships. It handles graph directionality and signed nature, capturing complex structures.
- SIHG [32]: SIHG combines the principle of information maximization and the advantages of hyperbolic geometric space.
- DINES [27]: DINES provides a novel method to handle the problems of signed networks. By learning decoupled node representations, it can understand and predict the complex relationships in the network more deeply.
- HEI [18]: HEI provides a novel method to handle the link prediction problem of treelike networks. It calculates the association between nodes based on the power of the difference in node degrees. This method can effectively predict missing links in highly sparse or treelike networks.
- TreeLSTM [16]: The user-subject symbol bipartite network is transformed into a tree structure with “user as root and subject as child node”, and the hierarchical position features are captured by LSTM.

### Experiment results

After completing the above introductions, we will officially start to verify and evaluate the TSGAT model. This paper conducts user reposting behavior prediction experiments and user reposting polarity prediction experiments to respectively evaluate the model’s ability to predict whether a reposting relationship occurs between users and whether the reposting relationship between users is positive or negative.

#### User retweet behavior prediction experiment.

The indicators of the user reposting behavior prediction experiment are shown in Tables 3 and 4, and the highest value in each row is bolded. This experiment mainly predicts the probability of new reposting relationships occurring between users who do not have reposting relationships, without considering the polarity of reposting relationships. Therefore, generally speaking, the SGCN and SiGAT models with relatively simple structures have poor prediction results on the datasets. The SDGNN, SIHG, and DINES models, due to considering more user and network features and learning user attributes and network structures of social networks to predict reposting behaviors, have certain improvements in both indicators on the datasets. Among them, the SDGNN model learns neighbor information in the network through a multichannel attention mechanism and performs the best among the above models. The TSGAT of this paper not only uses the attention mechanism to aggregate multiple neighbor information of users but also considers the topical stance features.

**Table 3:** F1 Values of user reposting behavior prediction experiment results.

Dataset	SGCN	SiGAT	SDGNN	SIHG	DINES	TreeLSTM	TSGAT
Taiwan Elections	0.832	0.845	0.891	0.882	0.892	0.774	**0.898**
US Presidential Election	0.875	0.880	0.912	0.895	0.908	0.777	**0.934**
Israeli Palestinian Conflict	0.849	0.829	0.907	0.883	0.891	0.688	**0.910**
Wahaha	0.889	0.899	0.907	0.893	0.891	0.743	**0.936**
Mayday Lip-synch	0.833	0.841	0.862	0.870	0.865	0.691	**0.894**
Jackey Xue Screen-shot	0.802	0.839	0.866	0.857	0.856	0.743	**0.937**

**Table 4:** AUC Values of user reposting behavior prediction experiment results.

Dataset	SGCN	SiGAT	SDGNN	SIHG	DINES	TreeLSTM	HEI	TSGAT
Taiwan Election	0.765	0.752	0.809	0.811	0.816	**0.896**	0.893	0.882
US Presidential Election	0.785	0.793	0.881	0.851	0.809	0.898	0.873	**0.909**
Israeli Palestinian Conflict	0.806	0.735	**0.877**	0.869	0.813	0.784	0.875	0.873
Wahaha	0.837	0.834	0.894	0.871	0.844	0.842	**0.964**	0.933
Mayday Lip-synch	0.799	0.763	0.822	0.817	0.803	0.760	0.834	**0.888**
Jackey Xue Screen-shot	0.794	0.772	0.839	0.816	0.802	0.852	0.849	**0.868**

HEI obtains the highest AUC on Wahaha, confirming that simple tree features are powerful when cascades are shallow.TreeLSTM performs competitively on small trees but degrades on large cascades (Mayday Lip-synch, Israeli-Palestinian) because it only sees local parent-child paths.

The TSGAT in this paper employs an attention mechanism to aggregate multiple neighbor information of users and also takes topical stance features into account. TSGAT is superior to other methods in four datasets of six datasets, and also provides edge polarity which the propagation chain prediction method based on tree structure does not have this ability. The combination of capturing rich neighbor interactions via the attention mechanism and integrating topical stance features allows TSGAT to effectively grasp the complex patterns of user reposting behavior, thereby demonstrating good predictive performance.

#### User retweet polarity prediction experiment.

The indicators of the user reposting polarity prediction experiment are shown in Tables 5 and 6, with the highest value in each row bolded. Different from the user reposting behavior prediction experiment, the user reposting polarity prediction experiment focuses on predicting the positive or negative polarity of the existing reposting relationships between users and pays more attention to the sign polarity of network edges. Therefore, the SGCN and SiGAT models, which mainly handle the sign nature of the network structure, can both achieve good results on the datasets. Among them, the SiGAT model uses an attention mechanism to dynamically weight the sign information of neighboring nodes and achieves the best results in terms of the AUC indicator on the Taiwan “Election” dataset and the F1 indicator on the Re: HH dataset. In addition, the TSGAT model of this paper achieves the best indicators in experiments on other datasets.

**Table 5:** F1 Values of user reposting polarity prediction experiment results.

Dataset	SGCN	SiGAT	SDGNN	SIHG	DINES	TSGAT
Taiwan Election	0.814	0.849	0.847	0.818	0.766	**0.859**
US Presidential Election	0.802	0.769	0.804	0.757	0.844	**0.877**
Israeli Palestinian Conflict	0.801	0.770	0.792	0.745	0.791	**0.832**
Wahaha	0.850	**0.868**	0.815	0.851	0.848	0.855
Mayday Lip-syncing	0.825	0.831	0.828	0.810	0.779	**0.860**
Jacky Xue Screen-shot	0.808	0.847	0.846	0.806	0.790	**0.852**

**Table 6:** AUC Values of user reposting polarity prediction experiment results.

Dataset	SGCN	SiGAT	SDGNN	SIHG	DINES	TSGAT
Taiwan Election	0.801	**0.827**	0.797	0.737	0.728	0.802
US Presidential Election	0.780	0.735	0.732	0.722	0.787	**0.790**
Israeli Palestinian Conflict	0.759	0.734	0.775	0.693	0.755	**0.822**
Wahaha	0.813	0.821	0.776	0.740	0.785	**0.823**
Mayday Lip-syncing	0.805	0.793	0.805	0.716	0.736	**0.808**
Jacky Xue Screen-shot	0.802	0.817	0.818	0.709	0.750	**0.824**

#### Ablation experiment.

In the ablation experiment, this paper tests the influence of topical stance features and the attention mechanism on user reposting prediction. The following situations are considered:

- TSGAT-S: A model that only fuses topical stances and does not incorporate the attention mechanism.
- TSGAT-A: A model that only incorporates the attention mechanism and does not consider topical stances.

The user reposting polarity prediction experiment based on the AUC index is carried out on the social network dataset, as shown in Table 7. From the results in the table, it can be seen that both the topical stance feature and the attention mechanism can effectively improve the prediction performance of the model. The models that consider only the topical stance feature or only the attention mechanism show a decrease in the AUC index on the six datasets, and the overall index results have little difference, indicating that both the topical stance feature and the attention mechanism contribute to the improvement of the experimental performance.

**Table 7:** Results of user reposting polarity prediction ablation experiment.

Dataset	TSGAT-S	TSGAT-A	TSGAT
Taiwan Election	0.774	0.759	**0.802**
US Presidential Election	0.746	0.735	**0.790**
Israeli Palestinian Conflict	0.759	0.774	**0.822**
Re: HH	0.783	0.782	**0.823**
Mayday Lip-syncing	0.780	0.773	**0.808**
Joker Xue Screen-shot	0.802	0.797	**0.824**

Moreover, the TSGAT model that incorporates both the topical stance feature and the attention mechanism achieves the best prediction results on all six datasets, which shows that considering the users’ stance features in the global network and aggregating the positive and negative polarity information of neighbors can significantly improve the user reposting prediction effect.

This chapter proposes a graph attention neural network user reposting prediction model (TSGAT) that integrates topical stances. It analyzes the topical stance features using the relationships between users, and then uses the graph attention neural network to learn the information of users’ neighbors with positive and negative polarities respectively. By comprehensively considering users’ neighbor information and network stance information and fusing them, a low dimensional representation vector of users is obtained, so as to perform user reposting prediction. Finally, two tasks, namely user reposting behavior prediction and user reposting polarity prediction, are used to verify the effectiveness of the method.

## Discussion

This paper’s research on social network user reposting prediction still has room for improvement, and future work can be carried out in several aspects.

One critical extension is to deepen the analysis of adaptability between two key elements: the dynamic evolution laws of tree-like propagation structures investigated in Shang et al. [18] and the static user network constructed in this paper. Exploring how to integrate short-term propagation segment features into our proposed long-term user relationship prediction framework would be highly valuable. These features include path overlap and hierarchical distance, both derived from tree-like propagation chains. This in-depth correlation analysis not only clarifies the technical complementarity between the two modeling paradigms but also enables a more holistic characterization of retweet dynamics. It further unifies insights into immediate content-specific propagation and enduring user stance associations.

Due to the limitations of our data sources, this study only conducts user retweet prediction research on data from Weibo and Twitter platforms. However, other online social platforms such as Facebook, Zhihu, and Tieba also have similar user-topic mechanisms, where users can discuss and spread related content to form topic stances. The topic stance representation and retweet prediction methods designed in this paper are not fully applicable to other social media platforms. For these platforms, it is necessary to consider their unique characteristics, user attributes, and mechanisms for topic discussion and retweeting. A comprehensive analysis of the factors influencing user retweeting and extending our research to other social platforms will be one of the focuses of future work.

The method designed in this paper only considers the binary polarity of network edges. In real-world scenarios, however, the emotions between users and their viewpoints on hot topics are often continuous and change over time. More precise analyses of user sentiment and topic stance are therefore needed, representing them as weighted positive and negative edges in the network and incorporating temporal factors to capture the evolution of user emotions and opinions. Hence, future research could focus on more detailed analysis and measurement of user sentiment and stance.

The method designed in this paper takes into account both the emotional polarity between users and topic stance features. On this basis, the network characteristics considered can be further expanded. For example, more user features such as basic information and personal preferences on social platforms can be included. Additionally, association information between users, such as following relationships, reports, or blocklists on platforms like Weibo and Twitter, can be considered. Moreover, in real-world scenarios, post forwarding on social media platforms is influenced by various factors such as platform policies and external social events. The impact of such information on user forwarding prediction can be further analyzed.

Moreover, the model structure of TSGAT needs to be optimized to reduce computational complexity, adapting to the efficient training requirements of large scale social networks.

## Conclusion

This paper focuses on the issue of social network user reposting prediction. Given the lack of user relationship polarity in real scenarios and the insufficiency of traditional methods in utilizing topic node information and considering global network features, the paper puts forward corresponding solutions. A topical stance representation method based on topic node weighting (TNTSR) is proposed, which combines node features and network balance structure to weight topic nodes, reconstruct user relationship networks, and improve the accuracy of topical stance characterization, with its effectiveness verified by community detection experiments. Additionally, a graph attention neural network model (TSGAT) integrating topical stances is designed, which aggregates users’ positive and negative polarity neighbor information and combines topical stance features to generate user representation vectors, achieving better performance in user reposting behavior and polarity prediction tasks, as validated by experiments on datasets from platforms like Twitter and Weibo.

The ablation experiments verify that both topical stance features and attention mechanisms are critical: topical stances capture the topic-specific attitudes of users, while attention mechanisms differentiate the contributions of positive/negative neighbors, enhancing representation learning. Compared to baselines (e.g., SGCN, SiGAT), TSGAT better models the ‘user-topic-user’ ternary relationship and handles multisource heterogeneous information (user features, topic semantics, edge polarities), explaining its higher accuracy.
